# Airway Changes After Sleep Apnea Surgery Using Drug‐Induced Sedation Endoscopy: A Systematic Review and Meta‐analysis

**DOI:** 10.1002/ohn.70028

**Published:** 2025-11-12

**Authors:** Pranav A. Patel, C. Cooper Munhall, Carter D. Smith, Mohamed Faisal Kassir, Emily A. Brennan, Badr Ibrahim, Shaun A. Nguyen, Mohamed Abdelwahab

**Affiliations:** ^1^ Sleep Surgery Division, Department of Otolaryngology–Head and Neck Surgery Medical University of South Carolina Charleston South Carolina USA; ^2^ MUSC Libraries Medical University of South Carolina Charleston South Carolina USA; ^3^ Department of Sleep Surgery Division, Department of Otolaryngology–Head and Neck Surgery University of Montreal Montreal Quebec Canada; ^4^ Department of Oral and Maxillofacial Surgery, School of Dentistry Medical University of South Carolina Charleston South Carolina USA

**Keywords:** DISE, maxillomandibular advancement, MMA, nasal surgery, obstructive sleep apnea, OSA, palate surgery, sedation endoscopy, sleep endoscopy

## Abstract

**Objective:**

To characterize drug‐induced sleep endoscopy as a method of evaluating the efficacy of sleep apnea surgical procedures at different airway sites.

**Data Sources:**

PubMed, Scopus, and CINAHL.

**Review Methods:**

Two independent investigators selected studies on sleep apnea surgeries with presurgical and postsurgical drug‐induced sedation endoscopy (DISE) evaluations. Primary research studies were included, and data that overlapped with another study were excluded. Investigators performed data extraction, quality rating, and risk‐of‐bias assessment using the ROBINS‐I tool. Data were analyzed using a comparison of proportions and means.

**Results:**

Ultimately, 10 studies (N = 320) were included, with 81.2% male (95% CI: 72.0‐88.9), and an average age of 46.8. Significant reductions in VOTE (velum, oropharynx, tongue, and epiglottis) scoring were observed in postsurgical DISE findings at the level of the velum and oropharynx within the palate group (*P* < .05), and at the velum, oropharynx, and tongue within the maxillomandibular advancement (MMA) group. There were also accompanying reductions in traditional measures such as apnea‐hypopnea index (AHI) and Epworth sleepiness score (ESS). No significant reductions in VOTE scores at any level were observed within the nasal surgery group.

**Conclusion:**

DISE allows clinicians a nuanced view in postoperative changes in upper airway collapse patterns and may provide a unique perspective in the evaluation of surgical outcomes in sleep apnea. Further research should be done to correlate these findings to existing outcome measures.

## Graphical abstract

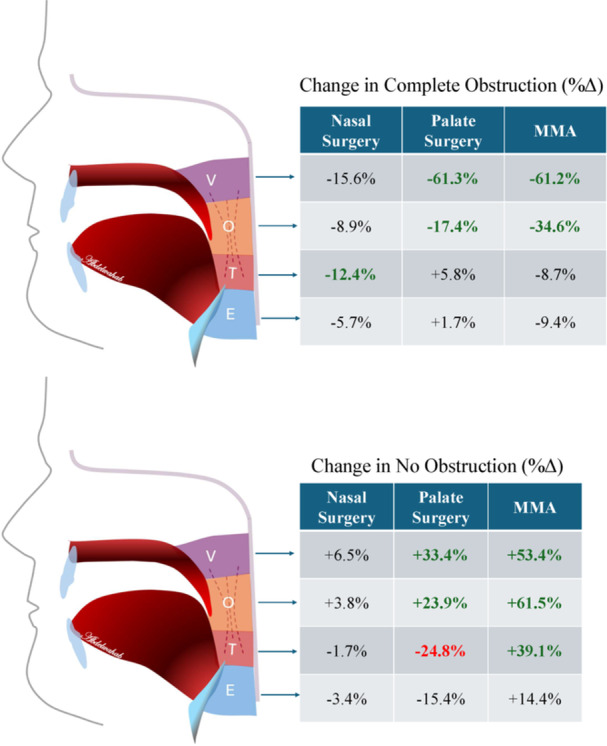

Obstructive sleep apnea (OSA) poses a substantial medical challenge, with repetitive upper airway collapse during sleep leading to fragmented sleep and subsequent hypoxemia. This extends beyond sleep disturbances, with previous literature showing associations with cardiovascular issues, metabolic disorders, cognitive impairments, and increased healthcare utilization, as well as a significant decrease in quality of life and increased mortality rates.^1^

Despite a predictable array of symptoms, the etiology of OSA is a multifactorial interplay of airway anatomy and physiology, including soft tissue airway collapse, pharyngeal muscle contractility, arousal threshold, and stability of the respiratory control system, among various other factors.^2^ While airway collapsibility has been shown to be significantly different between subjects, various patterns of collapse have been identified.^2^, ^3^, ^4^ Most OSA patients demonstrate retropalatal collapse. Patients with severe OSA with considerable nocturnal hypoxemia tend to exhibit greater lateral wall collapse than patients with milder OSA.^5^, ^6^ The variability in severity and type of airway collapse patterns seen in patients with moderate‐to‐severe OSA likely has important implications for treatment, which ideally focuses on the primary anatomical cause.^2^, ^3^

One diagnostic tool for assessing the dynamic changes in the upper airway is drug‐induced sedation endoscopy (DISE), which allows direct visualization of collapse patterns and may allow for the development of anatomically targeted interventions to address the specific site of collapse.^7^, ^8^, ^9^, ^10^ In comparison to traditional awake endoscopy, DISE provides a dynamic assessment that mimics natural sleep conditions to some extent, with the hope of offering a more accurate depiction of the airway phenotype.^6^ Recent evidence evaluating the correlation between preoperative DISE and surgical success suggests mixed results, but points to an overall advantage of preoperative DISE over cheaper, less invasive assessment methods.^11^, ^12^

For patients that fail standard medical therapy (CPAP), surgical options for OSA are considered. These options include soft tissue and skeletal procedures to the upper airway anatomy, and in our study, are separated into three distinct groups: maxillomandibular advancement (MMA), palate surgery, hypoglossal nerve stimualtion, and nasal surgery. Dynamic airway changes during sleep are highly variable; therefore, understanding the level and pattern of airway obstruction is crucial in determining targeted surgical interventions and predicting outcomes.^13^

MMA is a global reconstructive airway procedure that advances both the maxilla and mandible, resulting in an expansion of both the skeletal structure along with the connected soft tissue structure of the upper airway.^14^ Palate surgery is a relatively broad category encompassing a variety of nuanced surgical techniques and practices that address the soft palate and surrounding tissues. Palatopharyngoplasty (PPP) entails tonsillectomy followed by suture expansion of the airway, whereas uvulopalatopharyngoplasty (UPPP) also includes the removal of the uvula and part of the palate. A multitude of techniques have been described including lateral, sphincter, expansion, barbed, and preservation pharyngoplasty, each with a specific technique though similar underlying concept.^15^, ^16^, ^17^, ^18^ Each of these aims to widen the airway by altering the anatomy of the soft palate and, to some extent, the lateral pharyngeal wall (LPW).

Functional septoturbinoplasty or septorhinoplasty entails repositioning or removing deviated sections of the nasal septum to improve alignment and reducing the turbinates to alleviate nasal congestion and enhance airflow with or without nasal valve collapse reconstruction. Hypoglossal nerve stimulation surgery (HGNS) is a relatively new procedure where the muscle response is augmented during inhalation supporting the tongue base primarily. This can improve the retropalatal space collapse as well.^19^

While DISE has been validated as an effective tool for preoperative planning to improve surgical outcomes, little is known about the specific impact of different surgical techniques on the patterns of airway collapse or the correlation of these changes in OSA severity. A possible cause of the mixed results is a poor understanding of the postoperative changes in airway dynamics and the specific alterations in DISE findings following various surgical interventions. Understanding postoperative DISE changes can provide feedback on surgical techniques, optimizing patient outcomes, and potentially identifying predictors of success or failure for each procedure. For example, DISE can help in patient selection for HGNS, where complete concentric collapse is an exclusion criterion and tongue base collapse is a prognostic factor.^20^ This systematic review aims to bridge this knowledge gap by exploring the dynamic alterations in collapse patterns observed on DISE in patients after surgery for OSA. By examining the postoperative DISE findings, we aspire to offer insights into the anatomical and functional changes in the upper airway following various surgical interventions and their implications fo OSA phenotypic management. We hypothesize that each procedure can have a primary site(s) where it exerts its primary action using postoperative DISE.

## Methods

### Search Strategy

The systematic review was conducted according to the Preferred Reporting Items for Systematic Review and Meta‐analyses (PRISMA) Statement. To identify studies for inclusion, a librarian (E.A.B.) developed detailed search strategies in PubMed (US National Library of Medicine, National Institutes of Health), Scopus (Elsevier), and CINAHL Complete (EBSCOhost). The databases were searched from inception through November 6, 2023. The search strategies used a combination of subject headings (eg, MeSH in PubMed) and keywords for the concepts of postsurgical drug‐induced sleep endoscopy and OSA. The PubMed search strategy was modified for the other two databases, replacing MeSH terms with appropriate subject headings, when available, and maintaining similar keywords. English language filters were applied. The search strategies for each database are detailed in Supplemental Appendix 1, available online. To identify additional articles, the reference lists of included articles were hand searched along with cited articles.

Primary outcomes were changes that occur at each level of the VOTE (velum, oropharynx, tongue, and epiglottis) scoring during the DISE. This was followed by calculating the percentage of resolution of any obstruction at each site for each procedure as well as the percentage of management of complete obstruction. Secondary outcomes were evaluating the apnea‐hypopnea index (AHI), oxygen desaturation index (ODI), and Epworth sleepiness score (ESS) if reported with each procedure. Inclusion criteria included sleep surgery for OSA (MMA, palate surgery, hypoglossal nerve stimulator [HGNS], and nasal surgery), preoperative and postoperative DISE scoring, subjective (ESS, Sleep Apnea Quality of Life Index) or objective (AHI, ODI) surgical outcomes, primary research studies (clinical trials, observational studies), English language, and participant age older than 18 years. While the overall impact of such interventions on objective improvements in OSA is unclear, the nasal surgery group was included as previous literature has demonstrated improved sleep quality and decreased OSA symptoms postoperatively.^21^, ^22^ To eliminate potential sources of sampling bias, included studies were reviewed to ensure that all participants systematically underwent DISE; that is, all participants were given this procedure regardless of postoperative residual OSA symptoms. Exclusion criteria included no surgical intervention for OSA, lack of postsurgical DISE or outcomes measures, non‐primary research, nonhuman studies, or non‐English language.

References were exported into the review management software, Covidence, for de‐duplication and study selection.^23^ Two reviewers (C.D.S., P.A.P.) independently screened titles and abstracts to determine eligibility. Conflicts were resolved by discussion and consensus. Following the same process, the two reviewers then independently screened full‐text articles with conflicts being resolved through discussion and consensus.

### Data Extraction

Demographic, surgical, and outcomes data were collected from the studies included. Of these, the degree of obstruction during DISE was extracted according to the VOTE criteria.^24^ Change in severity of OSA was recorded as changes in AHI and ESS scores. While other outcomes such as Sher's surgical success, oxygen saturation nadir, and time spent below 90% of oxygen saturation were extracted, the decision was made to utilize AHI, ODI, and ESS only, as these measures were the most frequently reported and are widely used clinically for assessing the severity of OSA.

### Statistical Analysis

Meta‐analysis of continuous measures (age, body mass index [BMI], AHI, ODI, and ESS) and meta‐analysis of proportions (gender, VOTE scores presurgery vs postoperatively) were performed using Comprehensive Meta‐Analysis version 4 (Biostat Inc). Meta‐analyses of mean difference (pre‐MMA vs post‐MMA) for AHI, ODI, and ESS were performed with Cochrane Review Manager (RevMan) version 5.4 (The Cochrane Collaboration 2020, United Kingdom). Each measure (mean, mean difference [Δ], proportion [%], and 95% confidence interval [CI]) was weighted according to the number of patients affected. As some studies reported the outcomes in median (range), the quantile estimation (QE) method was deployed to calculate the pooled estimates.^25^, ^26^ Heterogeneity among studies was assessed using *χ* ^2^ and *I* ^2^ statistics with fixed effects (*I* ^2^ < 50%) and random effects (*I* ^2^ > 50%). In addition, a comparison of proportions, expressed as difference (Δ) and 95% CI, was done to compare outcomes between the two groups. Potential publication bias was evaluated by visual inspection of the funnel plot and Egger's regression test, which statistically examines the asymmetry of the funnel plot.^27^, ^28^ A *P*‐value of <.05 was considered to indicate a significant difference for all statistical tests. Interpreting data was categorized into two main findings:

‐ The capability of the procedure to manage complete obstruction at each site; significant reduction in complete obstruction.
‐ The capability of the procedure to eliminate obstruction at each site; significant increase in no obstruction.

### Quality Assessment

Initially, all included studies were evaluated for their level of evidence using the Joanna‐Briggs Institute level of evidence scale.^29^ In addition, all studies were evaluated using the ROBINS‐I risk‐of‐bias tool for nonrandomized studies or interventions.^30^

## Results

### Overview of Search Strategy

**Figure 1:**
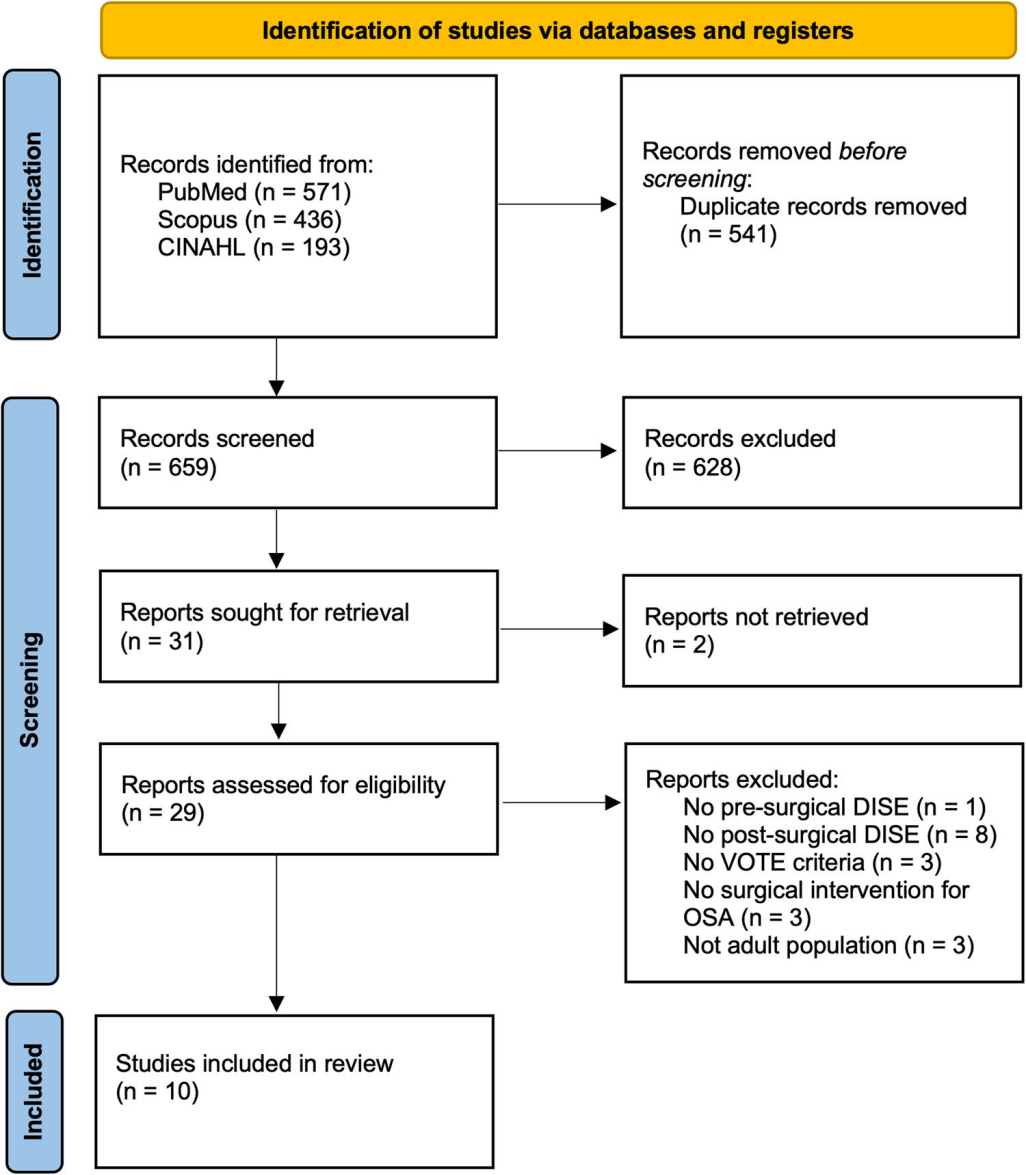
Preferred Reporting Items for Systematic Reviews and Meta‐analyses (PRISMA) diagram of included studies. DISE, drug‐induced sedation endoscopy; OSA, obstructive sleep apnea; VOTE, velum, oropharynx, tongue, and epiglottis.

The literature search revealed a total of 1200 studies, from which 541 duplicates were removed. Title and full‐text screening further reduced the included studies to 31 for full‐text review. A total of 10 studies were included in the final analysis (Figure 1), with one study included only as descriptive statistics. Table 1 provides a summary of included studies.

**Table 1:** Summary of Included Studies

Study	Study design	Country	LOE	Surgery type	Total patients (n)	Mean age (SD), y	Males (%)	Mean BMI (SD)	Mean pre‐op AHI (SD)	Outcomes analyzed
*MMA Group*										
Kastoer 2020	Prospective cohort study	Belgium	3e	MMA	14	51 (7)	57.14	25.6 (3.7)	40.2 (25.6)	AHI, ESS, ODI, min <90 SpO_2_
Liu 2016	Retrospective cohort study	United States	3e	MMA	20	44 (12)	85.00	27.4 (4.6)	53.6 (26.6)	AHI, SaO_2_ min, ESS, ODI
*Palate Surgery Group*										
Chiu 2021	Prospective cohort study	Taiwan	3e	PPP with tonsillectomy	34	46.4 (11.4)	82.35	27.9 (4.1)	40.6 (23.2)	AHI, SaO_2_ min
Hasselbacher 2018	Retrospective cohort study	Germany	3e	UPPP with tonsillectomy	15	47.3 (12.4)	100.00	30.9 (4.6)	34.7 (16.2)	AHI, ODI
Liu 2020	Prospective cohort study	United States	3e	PPP with tonsillectomy	12	68.2 (7.9)	75.00	30.5 (3.5)	54 (16.6)	AHI
Nikisha 2022	Prospective cross‐sectional	India	3e	Modified UPPP with tonsillectomy	129	44.2 (6.8)	63.57	32.63 (3.5)	33.93 (7.8)	AHI, SaO_2_ min, ESS, min <90 SpO_2_
Weidenbecher 2022	Retrospective cohort study	United States	3e	Expansion pharyngoplasty	20	56 (10)	70.00	30.7 (3.3)	53.9 (13.1)	AHI, ESS
*Nasal Surgery Group*										
Khode 2023	Prospective cohort study	UAE	3e	Functional septorhinoplasty with/without nasal valve suspension suture	32	38.88 (10.1)	93.75	28.7 (3.7)	45.81 (28.9)	AHI, ESS
Victores 2012	Retrospective cohort study	United States	3e	Functional septorhinoplasty	24	44.8 (13.9)	79.17	30.3 (5.9)	27.3 (18.1)	AHI, ESS
*INSPIRE Group*										
Heiser 2017	Retrospective cohort study	Germany	3e	Inspire (HGNS)	20	57 (12)	100.00	28.1 (13.1)	28.9 (7.6)	AHI, ESS

Abbreviations: AHI, apnea‐hypopnea index; BMI, body mass index; ESS, Epworth sleepiness score; HGNS, hypoglossal nerve stimulation surgery; LOE, level of evidence; MMA, maxillomandibular advancement; ODI, oxygen desaturation index; PPP, palatopharyngoplasty; UPPP, uvulopalatopharyngoplasty.

### Overview of Included Studies

Across all included studies, there were a total of 320 patients, with 81.2% male (95% CI: 72.0‐88.9). The average age was 45.1 (range: 31‐81) years. The mean BMI was 29.00 (±1.1), and the mean preoperative AHI was 38.56 (±5.69). Preoperative BMI for all surgical groups was evaluated and not found to be significantly different when compared among palate surgery, nasal surgery, and hypoglossal nerve stimulator groups. The only significant difference in preoperative BMI was observed between the palate and MMA surgical groups. The studies exhibited variability in their inclusion criteria, with the most prominent factors being CPAP failure, absence of prior surgical intervention for OSA, and moderate‐severe OSA (AHI > 15). All studies included in this review were a Joanna‐Briggs level of evidence of 3e. Studies included for analysis were published from 2012 to 2023 and originated from six different countries.

### Risk of Bias

**Figure 2:**
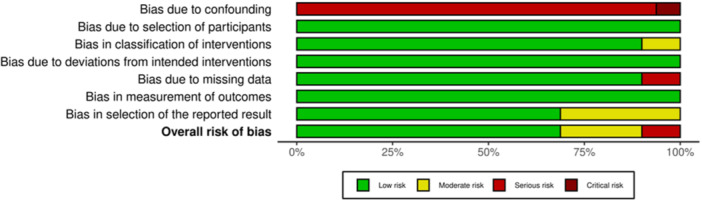
ROBINS‐I risk‐of‐bias assessment tool.

Critical appraisal of nonrandomized studies (Figure 2) indicated an overall acceptably low risk of bias with potential sources of bias being most pronounced from bias due to missing data and overall risk of bias. A funnel plot with Egger's test (3.08; 95% CI: −0.78 to 6.93; *P* = .10) demonstrated that all 10 studies were found within the funnel, suggesting low publication bias (Supplemental Figure S1, available online).

### Summary of Results

#### MMA Surgery

Two studies were included in the MMA group, with a total of 34 patients, with an average age of 47.8 ± 3.5 years.^5^, ^31^ Demographics and clinical characteristics were similar between the two studies. Comparison of proportions for presurgical and postsurgical outcomes on DISE demonstrated that the velum, oropharynx, and tongue were found to have the most significant reductions in obstruction, as shown in Table 2.

**Table 2:** Velum, Oropharynx, Tongue, and Epiglottis (VOTE) Classification, Maxillomandibular Surgery (N = 34)

Level of obstruction	Presurgical % (95% CI)	Presurgical *I* ^2^	Postsurgical % (95% CI)	Postsurgical *I* ^2^	% difference, (95% CI)	*P*‐value
*Velum*						
No obstruction	18 (2.44‐73.75)	92.25	71.8 (54.38‐85.48)	88.65	53.82 (30.74‐69.30)	<.0001
Partial obstruction	10.9 (3.01‐25.82)	0	22.3 (3.92‐50.01)	68.13	−11.43 (−29.13 to −6.76)	.21
Complete obstruction	70 (21.78‐99.44)	89.26	8.8 (0.71‐40.09)	81.52	−61.21 (−75.17 to −39.29)	<.0001
*Oropharynx*						
No obstruction	24.8 (0.02‐73.46)	89.37	86.3 (70.74‐95.45)	0	61.49 (38.98‐75.38)	<.0001
Partial obstruction	28.4 (1.86‐69.75)	84.88	13.7 (4.55‐29.26)	0	−14.76 (−33.24 to 4.86)	.14
Complete obstruction	50.0 (33.8‐66.2)	97.26	1.4 (0.00‐12.28)	0	−34.57 (−51.40 to −16.74)	.0003
*Tongue*						
No obstruction	19.3 (8.24‐33.55)	0	58.35 (40.77‐74.50)	0	39.08 (16.00‐56.92)	.001
Partial obstruction	58.4 (42.09‐73.73)	0	33.27 (18.51‐50.91)	0	25.13 (1.52‐45.16)	1
Complete obstruction	24.9 (12.38‐40.14)	0	16.28 (1.20‐43.57)	70.69	−8.65 (−27.42 to 10.77)	.38
*Epiglottis*						
No obstruction	57.3 (32.07‐80.67)	58.6	71.69 (43.22‐92.95)	67.99	14.38 (−8.11 to 34.99)	.22
Partial obstruction	22.4 (3.92‐50.01)	68.13	16.2 (6.08‐32.26)	0	−6.15 (−24.74 to 12.85)	.52
Complete obstruction	22.2 (10.07‐39.07)	81.52	12.76 (4.02‐28.12)	48.82	−9.39 (−27.39 to 9.09)	.31

Overall, there was a significant reduction in the percentage of patients with any visualized obstruction on preoperative and postoperative DISE findings following MMA at the velum (*P* < .0001), oropharynx (*P* < .0001), and tongue (*P* = .001) levels, as shown in Table 2. The epiglottis (*P* = .22) was not significantly impacted in the percentage of patients with postoperative presence of obstruction. There was also a significant reduction in the percentage of patients who had complete obstruction at the level of the velum (−61.21%, 95% CI: 39.29‐75.17, *P* < .0001) and oropharynx (−34.47, 95% CI: 16.74‐51.40, *P* = .0003). MMA showed significant elimination of obstruction at the LPW (the most, 62%), level of the velum (54%), and tongue base (39%). It was capable of reducing complete obstruction at the level of the velum (61%) and the LPW (35%).

**Figure 3:**
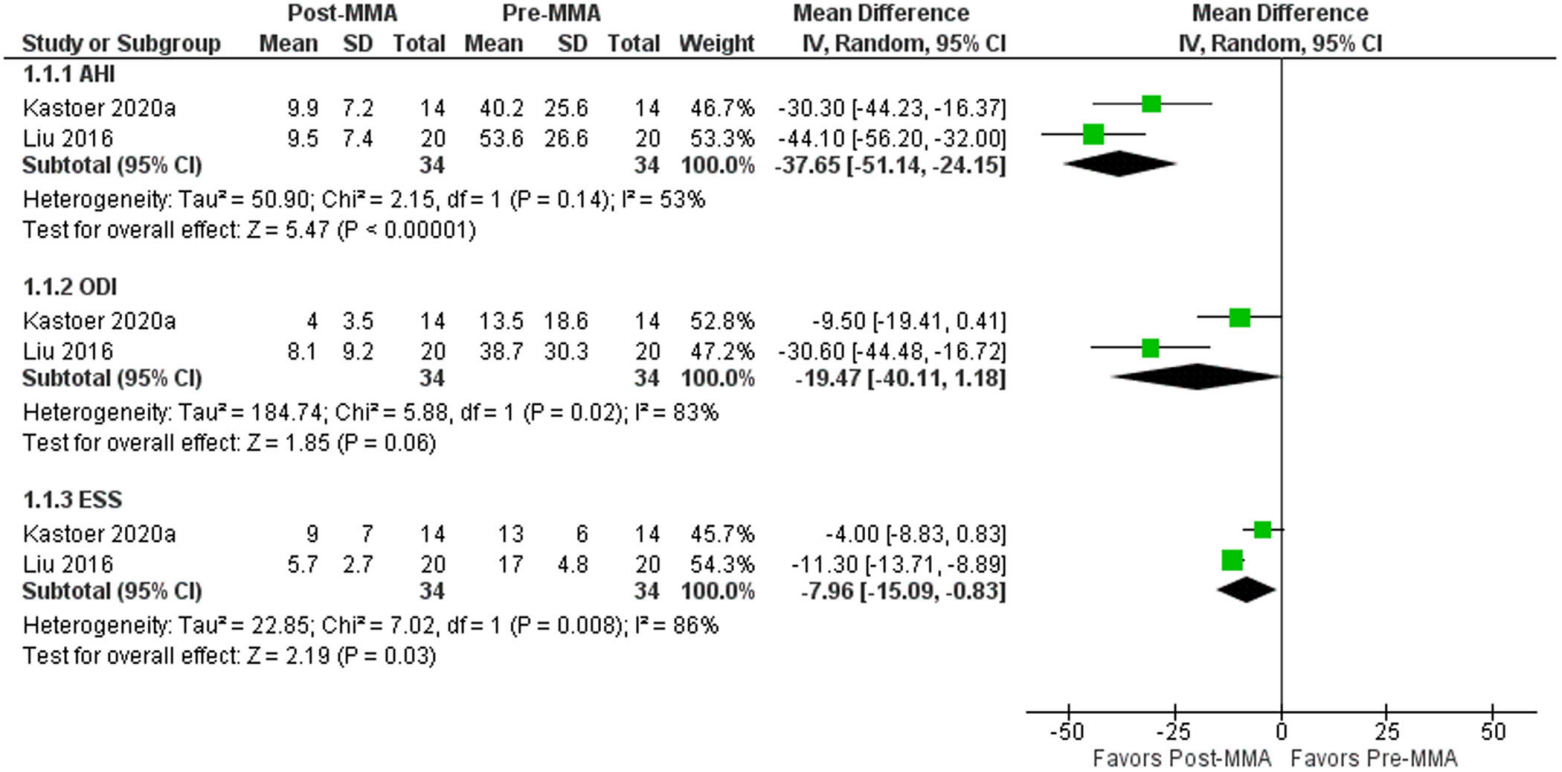
Forest plot of continuous variables with meta‐analysis of mean difference, maxillomandibular advancement (MMA) group. AHI, apnea‐hypopnea index; ESS, Epworth sleepiness score; ODI, oxygen desaturation index.

**Figure 4:**
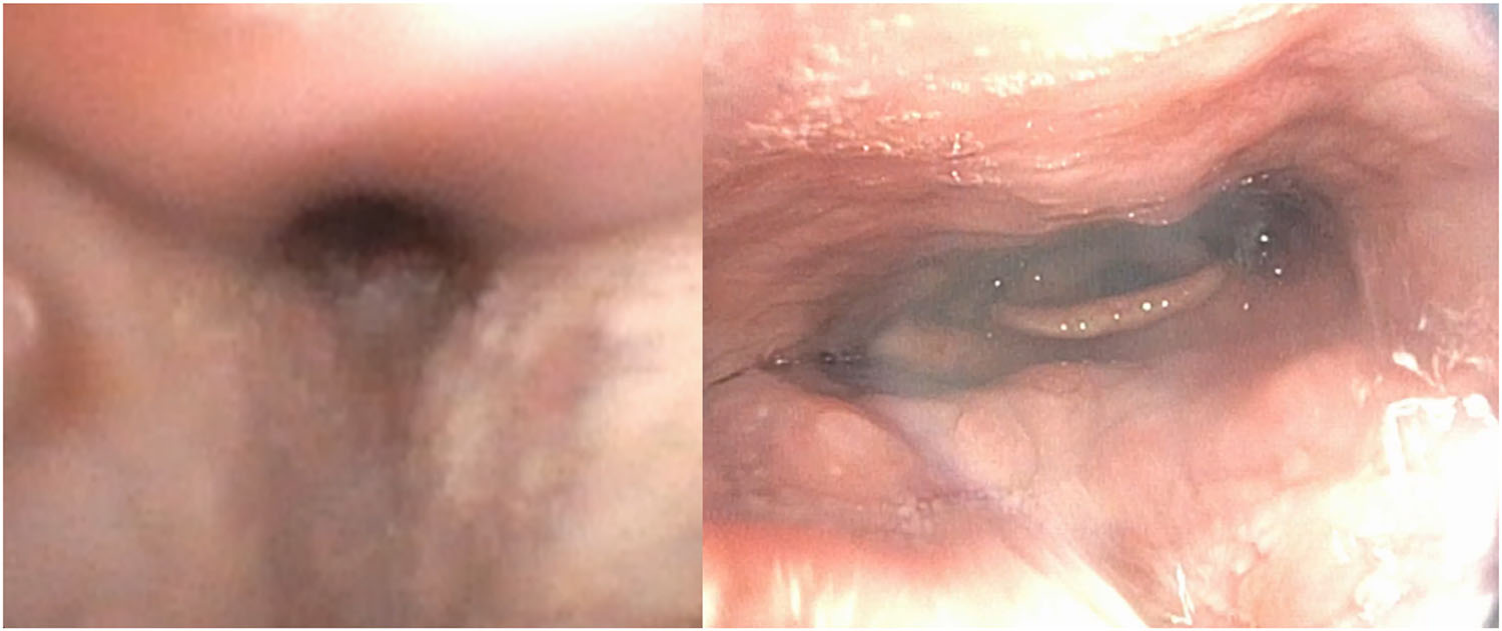
Airway comparison before and after maxillomandibular advancement surgery: pre‐surgery (left), the airway is significantly constricted. Post‐surgery (right), the airway shows a markedly increased diameter.

AHI (−37.65, 95% CI: −51.1 to −24.2, *P* < .00001) and ESS (−7.96, 95% CI: −15.1 to −0.8, *P* = .03) scores were also found to be significantly reduced postoperatively (Figure 3). An illustration of DISE findings before and following MMA surgery can be found in Figure 4.

#### Palate Surgery

Five studies were included in the palate surgery group, with a total of 210 patients, with an average age of 44.5 ± 0.6 years.^6^, ^32^, ^33^, ^34^, ^35^ Demographics and clinical characteristics were similar between the two studies. Comparison of proportions demonstrated a significant reduction in the percentage of patients with any obstruction preoperative and postoperative DISE findings at the velum (*P* < .0001), and oropharynx (*P* = .007), as shown in Table 3. Interestingly, there was a significant worsening of obstruction at the level of the tongue (*P* = .006), and no significance at the level of the epiglottis (*P* = .053).

**Table 3:** Velum, Oropharynx, Tongue, and Epiglottis (VOTE) Classification, Palate Surgery (N_velum, epiglottis_ = 210, N_oropharynx, tongue_ = 61)

Level of obstruction	Presurgical % (95% CI)	Presurgical *I* ^2^	Postsurgical % (95% CI)	Postsurgical *I* ^2^	% difference (95% CI)	*P*‐value
*Velum*						
No obstruction	0.5 (0.01‐2.58)	0	33.9 (5.83‐70.65)	95.67	33.38 (26.94‐40.03)	<.0001
Partial obstruction	0.5 (0.01‐2.58)	0	22.8 (2.19‐56.12)	94.93	22.30 (16.71‐28.44)	<.0001
Complete obstruction	99.5 (97.42‐99.99)	0	38.3 (15.44‐64.24)	90.95	−61.27 (−67.59 to 54.52)	<.0001
*Oropharynx*						
No obstruction	26.9 (0.62‐71.83)	92.26	50.8 (19.34‐81.95)	85.26	23.94 (6.64‐39.34)	.007
Partial obstruction	35 (12.95‐61.09)	75.87	30 (12.34‐51.58)	65.56	−4.93 (−20.98 to 11.49)	.56
Complete obstruction	33.4 (15.50‐54.26)	62.46	16 (4.26‐33.41)	59.7	−17.37 (−31.77 to −2.01)	.03
*Tongue*						
No obstruction	54 (41.07‐66.53)	41.47	29.2 (7.96‐57.04)	79.21	−24.77 (−40.25 to −7.28)	.006
Partial obstruction	28.7 (8.20‐55.52)	77.71	40.3 (11.31‐73.79)	85.84	11.62 (−5.17 to 27.54)	.1788
Complete obstruction	21.8 (12.47‐33.92)	37.98	27.7 (9.57‐50.75)	70.5	5.82 (−9.46 to 20.78)	.458
*Epiglottis*						
No obstruction	85.9 (43.1‐98.0)	86.2	69.3 (32.7‐91.3)	88.2	−15.43 (−30.13 to 0.19)	.0531
Partial obstruction	8.9 (1.6‐3.6)	82.09	15.7 (3.4‐50.1)	89.53	3.90 (−1.07 to 9.04)	.3994
Complete obstruction	8.1 (3.1‐19.9)	72.77	9.8 (4.5‐20.2)	75.1	1.7 (−9.2 to 12.7)	.0306

**Figure 5:**
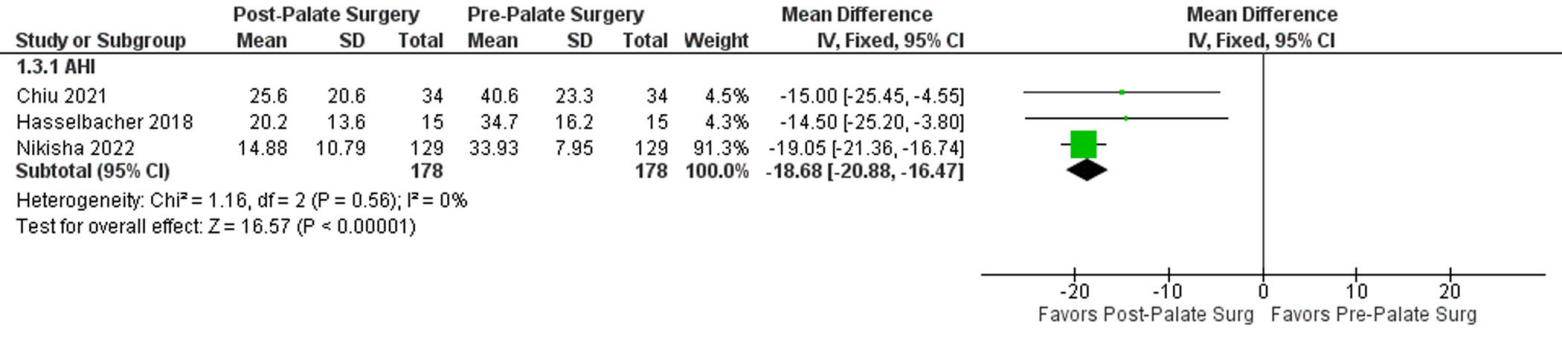
Forest plot of continuous variables, meta‐analysis of mean difference, palate surgery group. AHI, apnea‐hypopnea index.

Palate surgery showed significant elimination of obstruction at the level of the velum (the most, 66%) and LPW (17%). It could manage/improve complete obstruction at the level of the velum (33%) and the LPW (24%), and to a lesser extent, the epiglottis (5%). Meta‐analysis of mean difference showed a significant postoperative reduction in AHI (−18.68, 95% CI: −20.88 to −16.47, *P* < .00001), which is seen in Figure 5.

#### Nasal Surgery

Three studies were included in the nasal surgery group, with a total of 56 patients, with an average age of 46.8 ± 5.5.^19^, ^36^, ^37^ There were no significant preoperative and postoperative differences in level of obstruction, based on DISE findings: velum (*P* = .32), oropharynx (*P* = .69), tongue (*P* = .84), and epiglottis (*P* = .71) as shown in Table 4.

**Table 4:** Velum, Oropharynx, Tongue, and Epiglottis (VOTE) Classification, Nasal Surgery (N = 56)

Level of obstruction	Presurgical %, 95% CI	Presurgical *I* ^2^	Postsurgical, %	Postsurgical *I* ^2^	% difference (95% CI)	*P*‐value
*Velum*						
No obstruction	10.1 (3.72‐20.82)	0	16.5 (8.08‐28.62)	28.62	6.47 (−6.52 to 19.49)	.32
Partial obstruction	30.9 (19.45‐44.44)	0	37.2 (10.76‐68.67)	68.67	6.22 (−11.10 to 23.04)	.49
Complete obstruction	60.4 (46.65‐72.97)	0	44.8 (31.69‐58.40)	58.40	−15.59 (−32.57 to 2.81)	.10
*Oropharynx*						
No obstruction	46.4 (1.00‐97.09)	96	50.2 (4.48‐95.72)	95.72	3.80 (−14.27 to 21.52)	.69
Partial obstruction	33.5 (2.63‐77.06)	91.82	37.2 (10.76‐68.67)	68.67	3.67 (−13.70 to 20.74)	.69
Complete obstruction	18.9 (4.24‐40.42)	70.95	9.8 (1.07‐45.67)	45.67	−8.93 (−22.18 to 4.37)	.18
*Tongue*						
No obstruction	27.8 (12.13‐47.06)	57.99	26.2 (8.64‐49.06)	49.06	−1.67 (−17.79 to 14.57)	.84
Partial obstruction	57.3 (28.70‐83.49)	80.42	72.2 (52.94‐87.87)	87.87	14.83 (−2.81 to 31.26)	.10
Complete obstruction	15.1 (7.03‐26.89)	28.58	2.7 (0.20‐10.72)	10.72	−12.40 (−24.16 to −1.61)	.02
*Epiglottis*						
No obstruction	65.5 (51.89‐77.52)	0	62.1 (48.41‐74.52)	74.52	−3.42 (−20.58 to 14.02)	.71
Partial obstruction	22.2 (12.39‐35.11)	0	25.3 (6.24‐51.62)	51.62	3.04 (−12.66 to 18.56)	.71
Complete obstruction	13.5 (5.98‐25.09)	0	7.9 (1.15‐39.24)	39.24	−5.66 (−17.90 to 6.38)	.33

**Figure 6:**
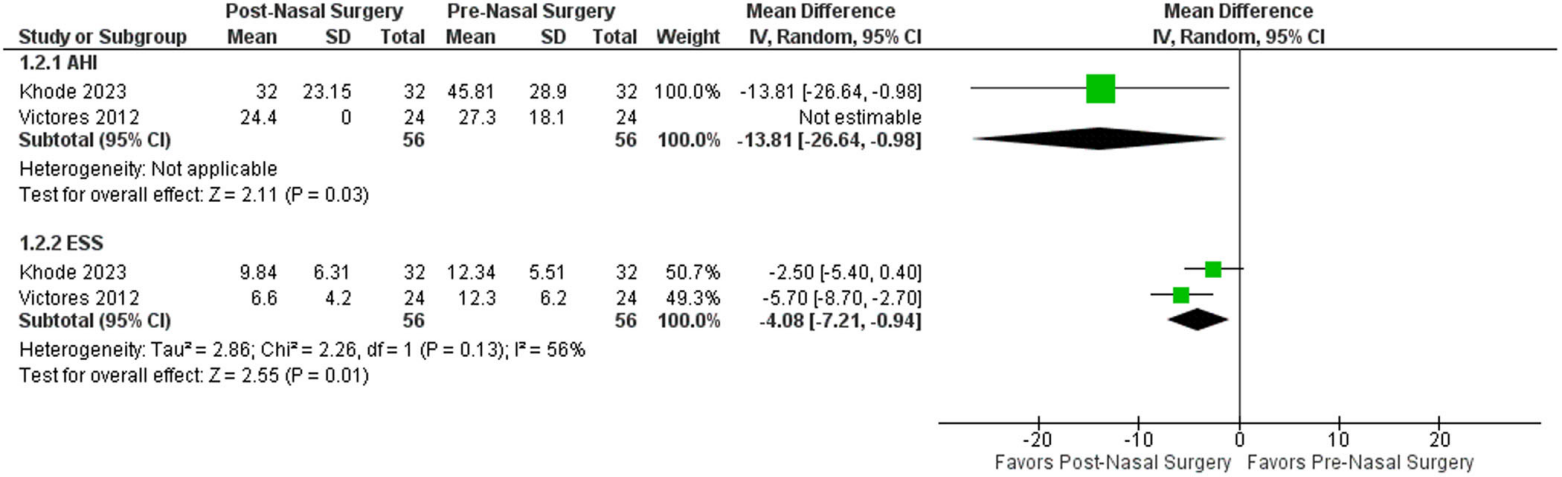
Forest plot of continuous variables with meta‐analysis of mean difference, nasal surgery group. AHI, apnea‐hypopnea index; ESS, Epworth sleepiness score.

Therefore, nasal surgery failed to significantly eliminate obstruction at any level. However, it could manage/reduce complete obstruction at the level of the tongue base in 12%. Comparison of continuous variables showed a significant postoperative reduction in AHI (−13.81, 95% CI: −26.64 to −0.98, *P* = .03) and ESS (−4.08, 95% CI: −7.21 to −0.94, *P* = .01), seen in Figure 6.

#### INSPIRE (HGNS) Surgery

One study was included in the INSPIRE group, with a total of 20 patients, with an average age of 57 ± 12.^19^ Preoperatively, most patients exhibited complete retropalatal (70%) and tongue base obstruction (80%), with no obstruction at the oropharynx (65%) and epiglottis (80%). Postoperatively, all patients were able to be successfully converted to no obstruction at the level of the velum (100%). Notably, there was no further postoperative data at the level of the oropharynx, tongue base, or epiglottis.

## Discussion

While the clinical presentation of OSA yields characteristic symptoms, the etiology is multifactorial and can have variable contributions from structural airway constriction, soft tissue collapse, pharyngeal muscle resting tone, to physiological factors like arousal threshold, and respiratory control system stability. In addition to a diverse array of potential causes, there is considerable variation even for a given etiologic factor.^2^ When assessing airway collapsibility, there are significant variations among individuals regarding the level and pattern of anatomic obstruction.^2^, ^3^, ^38^ DISE has been established as a validated method for identifying collapse patterns in patients during preoperative planning; however, there is limited information on the impact of specific sleep surgery interventions on postoperative collapse patterns observed during DISE.^8^, ^9^, ^10^ This systematic review and meta‐analysis aim to synthesize results from previous literature evaluating changes in DISE outcomes and airway collapse patterns following surgical interventions for OSA to provide insight on their direct airway impact.

### Group Surgical Outcomes

There were significant differences in preoperative and postoperative DISE findings at various anatomic levels for both the palate surgery and MMA groups. While both the palate and MMA groups had significant improvements in the percentage of patients with either no obstruction or lack of complete obstruction postoperatively, there were important nuances in terms of the degree of change and levels of obstruction on DISE. As expected, there were minimal, yet interesting significant differences on postoperative DISE for change in airway collapse or obstruction patterns within the nasal surgery group.

Nasal obstruction has been posited as a potential contributing factor to the development/severity of OSA, and thus, nasal surgery has been explored in addressing OSA.^39^, ^40^, ^41^, ^42^ Most studies of patients with OSA after nasal surgery show subjective improvement and potential for improved compliance with CPAP, but minimal improvement in objective outcomes and therefore limited efficacy as a standalone treatment for OSA.^39^, ^43^, ^44^ The nasal surgery group in this review showed no significant elimination of collapse levels. When evaluating its effect on the capability of managing complete obstruction, it showed significant improvement/reduction in complete tongue base obstruction. Nasal surgery significantly improved the ESS score, which is consistent with prior studies demonstrating improvement in subjective measures.^39^, ^43^, ^44^ While there was a significant reduction in AHI scores postoperatively, the OSA severity did not change overall; remaining in the moderate range in one of the included studies and in the severe range in the other.^36^, ^37^ Of note, neither study exhibited reductions in AHI meeting criteria for surgical success; reduction in AHI of >50% and AHI < 20 following surgical intervention.^45^ This is especially evident when comparing the reductions in AHI for MMA, HGNS, and palate surgery groups.^39^, ^43^, ^44^ Since nasal airflow is proximal to levels of obstruction visualized during a DISE (VOTE), it may not directly result in changes in the distal airway phenotype. However, breathing through the mouth provokes sleep disturbances, increases in stage 1 non rapid eye movement sleep (light sleep) duration, and the occurrence of obstructive apnea events; therefore, it is unsurprising that the nasal surgery group showed improvement in pre‐op AHI.^46^ This was evident as the tongue base demonstrated a significant improvement in postoperative obstruction, which is supported by the genioglossus reflex.^47^ Another possible explanation is the impact of nasal breathing on increased EMG response of the palatoglossus, which can in turn support the action of the genioglossus muscle.^48^ Ultimately, this review lends further credence to previous literature highlighting the potential synergy that nasal surgery can offer in multistage treatments for OSA.^37^, ^44^, ^49^ Of note, it is unclear if nasal valve surgery can contribute to improved outcomes as most articles include septoplasty and turbinate reduction only.

Given that both MMA and palate surgery have demonstrated efficacy in the treatment of OSA, it is important to consider preoperative phenotypic differences in airway collapse and expected postsurgical outcomes when determining appropriate surgical intervention. The results of this systematic review and meta‐analysis correlate with previous findings documenting improvement in velopharyngeal (velum) and, to some extent, the oropharyngeal (LPW) airway obstruction following palate surgery. Postsurgical stiffening of the lateral walls of the oropharynx for specific palate surgeries has been observed, such as lateral or expansion pharyngoplasty, resulting in decreased obstruction at this location.^18^, ^50^ Furthermore, other palate surgeries have demonstrated changes on postoperative DISE at the level of the velopharynx with elimination of obstruction or conversion of certain collapse patterns.^6^, ^32^ While isolated palate surgeries can have limited success in severe OSA or in patients with distal airway obstruction, the improvement noted above in collapse at the level of the velum and LPW can also improve overall treatment response or patient candidacy following other interventions such as MMA or HGNS.^51^, ^52^ Importantly for patient selection, favorable outcomes of isolated palate surgery have been associated with larger palatine tonsil size in addition to modified Mallampati tongue position 1 or 2 as having the best success rates.^53^, ^54^ These factors, in conjunction with the level and pattern of collapse visualized on DISE, may all be utilized to appropriately select patients who may experience the most benefit from certain palate procedures. In this review, it seems that palate surgery is most successful for patients with velum collapse on DISE and to a lesser extent in those with LPW collapse; however, data on the pattern of velum collapse were not determined.

As discussed previously, patients with severe OSA often demonstrate multilevel collapse, which may limit the efficacy of interventions that do not appreciably alter the corresponding airway level. While palate surgery does intuitively improve retropalatal collapse, it should not be expected to play any role in the improvement of retroglossal collapse. The findings of this review corroborate this understanding of palate surgery outcomes, with a surprising and notable significant increase in patients with visualized obstruction at the level of the tongue on DISE postoperatively. While it may seem unlikely that palate surgery worsens airway obstruction at the level of the tongue, the AHI still improved in the included studies. However, this is a finding that was reported previously by Shepard and Thawley using CT scans. When evaluating postoperative airways after UPPP, the hypopharyngeal segment corresponding to the retroglossal space decreased in cross‐sectional area by 23% to 25% after surgery.^55^ A possible technical explanation is that suturing the palatoglossus and the palatopharyngeus can result in a posterior displacement of the palatoglossus and hence the tongue base, resulting in further distal blockage that may contribute to failure of UPPP. Therefore, in our newer palate surgery techniques, “preservation” suturing usually takes place in an oblique fashion.^17^, ^56^, ^57^ It does pose an important consideration, however, for patients with retroglossal collapse patterns visualized on DISE in determining a surgical plan that may involve more than isolated palate surgery, hence HGNS. Although unlikely to be clinically meaningful given reductions in AHI in included studies, the significant increase seen in tongue base collapse following palate surgery could possibly be attributed to the proximal airway collapse relief unmasking distal airway collapse.

While palate surgery is generally limited to improvements in collapse patterns at the level of the LPW and none at the tongue base, this is not the case when MMA was evaluated postoperatively. The results of this meta‐analysis showed significant elimination of obstruction at the LPW predominantly followed by the velum in more than 50% of the cases, and lastly the tongue base in almost 40%. When evaluating the ability to manage complete obstruction at different sites, MMA was able to reduce complete obstruction mainly at the LPW, followed by the velum. Of note, the LPW collapse has been demonstrated as the main predictor of nocturnal hypoxemia and may be the reason behind the higher success rates after MMA.^58^ Larger improvements in rates of obstruction at multiple levels also correlated with the most dramatic reductions in AHI, with included studies for patients undergoing MMA all demonstrating postoperative AHI < 10 from severe ranges preoperatively. The only level at which MMA did not demonstrate significant changes in the presence of obstruction postoperatively was at the level of the epiglottis, which has previously been shown to detrimentally impact response rate and surgical success following MMA.^59^ While MMA therefore offers a more comprehensive, multilevel approach in the surgical treatment of OSA, there are still limitations to its efficacy at the level of the epiglottis. This serves as a further point of emphasis toward the appropriate workup of anatomic‐specific levels and patterns of obstruction through preoperative DISE. A more complete understanding of the ways in which preoperative DISE collapse patterns and levels of obstruction can be expected to change following specific surgical interventions can help guide surgical management toward anatomically targeted intervention. In addition to preoperative anatomic assessment, patient selection before consideration of MMA must also weigh the increased postoperative recovery time and additional risks involved with this more invasive procedure.

### Limitations

There are several limitations to this study, which may impact the generalizability of the data, including a lack of overall data and significant heterogeneity in reported outcome measures. First, when reviewing studies for inclusion and exclusion, there were limited studies evaluating the use of DISE as an assessment of postsurgical outcomes. When reviewing the studies that were included, the ROBINS‐I risk‐of‐bias assessment tool showed the lack of a control group as a significant limitation across all studies. Future prospective studies with nonsurgical control groups are warranted to further evaluate the use of DISE to assess postsurgical airway obstruction and collapse patterns. Although unclear if any clinically meaningful difference, the palate surgery group had a higher mean preoperative BMI than the MMA surgical group. Future prospective studies evaluating BMI as a potential confounding factor in rates of postoperative airway collapse among patients undergoing palate surgery may help better evaluate this as an independent risk factor. Our team is conducting other work in determining the impact of BMI on both palate and skeletal procedures. Although MMA demonstrated more profound improvements across various metrics, it is important to note that there were only two included studies with limited sample sizes to support these results. This is largely in part due to the lower surgical volume of MMA compared to other surgical modalities and serves as an important focus for future sleep surgery research to continue to expand upon these results. While other factors such as the subjectivity of DISE scoring were considered a low risk, including a nonsurgical control group would have increased the power of observed results. Future studies could also consider consensus or pooled scoring of DISE to reduce the risk of inter‐rater variability. Finally, the heterogeneity and lack of adequate study participants across studies in this review were too pronounced to conduct a true meta‐regression to evaluate the correlation between DISE findings and other objective and subjective outcome measures. The limitation of studies based on postoperative DISE results also limits the ability of this study to comment on overall reductions in AHI, ODI, and ESS. We also failed to include various patterns of airway collapse, for example, concentric versus anteroposterior (AP) patterns due to limitations in reported outcomes among studies.

## Conclusion

DISE has been a cornerstone in identifying airway collapse patterns and phenotypes preoperatively, though less consistently used in evaluating airway changes that occur following surgical intervention for OSA. This systematic review and meta‐analysis showed significant reductions in postoperative collapse patterns among both MMA and palate surgery groups, and partially at the tongue base in nasal surgery groups. Only one study reported postoperative results after HGNS featuring its impact on the velum. Future research should prioritize more detailed characterization of collapse patterns (eg, AP vs concentric vs LPW collapse) along with direct correlation of postoperative findings on DISE with the traditional measure of surgical success; namely, reductions in AHI and ODI. An improved understanding of the expected changes in postoperative anatomy and airway collapse phenotypes on DISE may allow surgeons to better tailor surgical plans to patient‐specific pathophysiology in effectively treating different OSA phenotypes with one or more procedures accordingly.

## Author Contributions

**Pranav A. Patel**, Made a substantial contribution to the concept or design, analysis, and interpretation of data. Drafted the primary manuscript and approved the final version to be published; **C. Cooper Munhall**, Substantial contribution to the interpretation of data. Drafted the primary manuscript and approved the final version to be published; **Carter D. Smith**, Made a substantial contribution to the design, analysis, and interpretation of data. Drafted the primary manuscript and approved the final version to be published; **Mohamed Faisal Kassir**, Made a substantial contribution to the design and interpretation of data. Revised the primary manuscript and approved the final version to be published; **Emily A. Brennan**, Made a substantial contribution to the concept or design of data; Revised the primary manuscript and approved the version to be published; **Badr Ibrahim**, Made a substantial contribution to the interpretation of data. Revised the primary manuscript and approved the version to be published; **Mohamed Abdelwahab**, Study design initation, made a substantial contribution to the concept or design, analysis, and interpretation of data. Revised the primary manuscript and approved the version to be published.

## Disclosures

### Competing interests

The authors declare no conflicts of interest.

### Funding source

None.

## Supporting information

Supporting Information.

Supporting Information.
