# Supplementary figures and images for: Airway Changes After Sleep Apnea Surgery Using Drug‐Induced Sedation Endoscopy: A Systematic Review and Meta‐analysis
Source: Otolaryngol Head Neck Surg. 2025 Nov 12;174(1):17–29. doi: 10.1002/ohn.70028 (PMC12794777; doi:10.1002/ohn.70028)

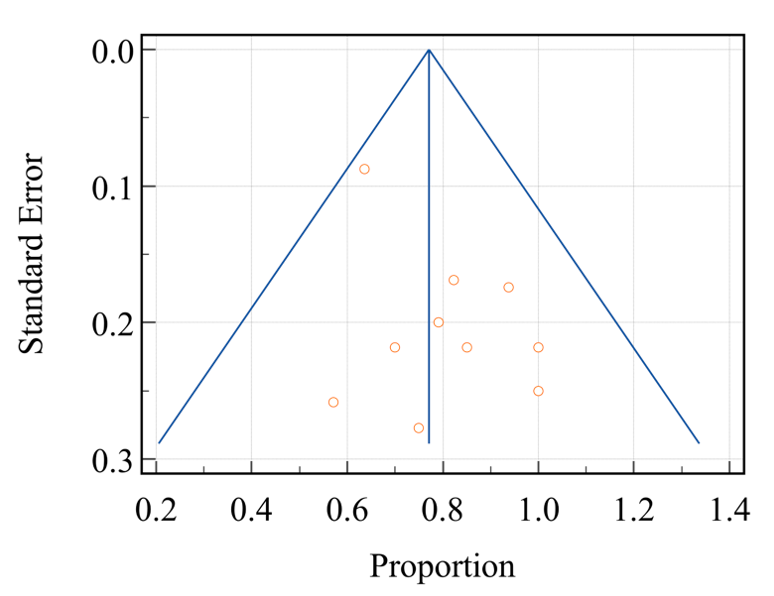

Supplement: Supplementary file 1 — Supporting Information. [file OHN-174-17-s002.png]
