# Supplementary material for: Airway Changes After Sleep Apnea Surgery Using Drug‐Induced Sedation Endoscopy: A Systematic Review and Meta‐analysis
Source: Otolaryngol Head Neck Surg. 2025 Nov 12;174(1):17–29. doi: 10.1002/ohn.70028 (PMC12794777; doi:10.1002/ohn.70028)
Supplement: Supplementary file 2 — Supporting Information. [file OHN-174-17-s001.docx]

**Detailed Search strategies:**

**PubMed (U.S. National Library of Medicine, National Institutes of Health):**

(dexmedetomidine[tw] OR DISE[tw] OR DISN[tw] OR drug-induced[tw] OR midazolam[tw] OR propofol[tw]) **AND** (anesthesia[tw] OR DISE[tw] OR DISN[tw] OR sedation[tw] OR sleep[tw]) **AND** ("Endoscopy"[Mesh] OR endosco*[ti] OR nasendoscop*[tw] OR nasoendoscop*[tw] OR DISE[tw] OR DISN[tw]) **AND** ("apnea hypopnea*"[tw] OR "apnoea hypopnea*"[tw] OR bimaxillary[tw] OR "double-jaw"[tw] OR "genioglossus advancement"[tiab:~2] OR GA[ti] OR “hyoid myotomy”[tw] OR “hyoid suspension”[tw] OR "hypoglossal nerve stimulat*"[tw] OR HSNS[ti] OR LAUP[ti] OR "Mandibular Advancement"[Mesh] OR mandibular[tw] OR “maxillomandibular advancement”[tw] OR MMA[ti] OR OSA[ti] OR "Sleep Apnea, Obstructive"[Mesh] OR "sleep apnea*"[tw] OR "sleep apnoea*"[tw] OR "sleep-disordered breathing"[tw] OR "sleep hypopnea*"[tw] OR uvulopalatopharyngoplast*[tw] OR UPPP[ti] OR UP3[ti] OR "upper airway stimulat*"[tw])

- Filters/limits: English
- Date searched: November 6, 2023
- # of records identified: 571

**Scopus (Elsevier) search strategy:**

**#1: TITLE:** (dexmedetomidine OR DISE OR DISN OR drug-induced OR midazolam OR propofol) **AND** (anesthesia OR DISE OR DISN OR sedation OR sleep) **AND** (**endosco*** OR nasendoscop* OR nasoendoscop* OR DISE OR DISN) **AND** ("apnea hypopne" OR "apnoea hypopnea"OR bimaxillary OR {double-jaw} OR "genioglossus advancement" OR **GA** OR “hyoid myotomy” OR “hyoid suspension” OR "hypoglossal nerve stimulation" OR **HSNS** OR **LAUP** OR mandibular OR “maxillomandibular advancement” OR **MMA** OR **OSA** OR "sleep apnea" OR "sleep apnoea" OR "sleep-disordered breathing" OR "sleep hypopnea" OR uvulopalatopharyngoplast* OR **UPPP** OR **UP3** OR "upper airway stimulation")

**#2: ABSTRACT:** (dexmedetomidine OR DISE OR DISN OR drug-induced OR midazolam OR propofol) **AND** (anesthesia OR DISE OR DISN OR sedation OR sleep) **AND** (nasendoscop* OR nasoendoscop* OR DISE OR DISN) **AND** ("apnea hypopne" OR "apnoea hypopnea"OR bimaxillary OR {double-jaw} OR "genioglossus advancement" OR “hyoid myotomy” OR “hyoid suspension” OR "hypoglossal nerve stimulation" OR mandibular OR “maxillomandibular advancement” OR "sleep apnea" OR "sleep apnoea" OR "sleep-disordered breathing" OR "sleep hypopnea" OR uvulopalatopharyngoplast* OR "upper airway stimulation")

**#3:** #1 OR #2

- Filters/limits: English
- Date searched: November 6, 2023
- # of records identified: 436

**CINAHL Complete (EBSCOhost) search strategy:**

(dexmedetomidine OR DISE OR DISN OR drug-induced OR midazolam OR propofol) **AND** (anesthesia OR DISE OR DISN OR sedation OR sleep) **AND** (MH "Endoscopy+" OR TI endosco* OR nasendoscop* OR nasoendoscop* OR DISE OR DISN) **AND** ("apnea hypopnea" OR "apnoea hypopnea" OR bimaxillary OR "double-jaw" OR "genioglossus advancement" OR TI GA OR “hyoid myotomy” OR “hyoid suspension” OR "hypoglossal nerve stimulation" OR TI HSNS OR TI LAUP OR mandibular OR “maxillomandibular advancement” OR TI MMA OR TI OSA OR MH "Sleep Apnea, Obstructive" OR "sleep apnea" OR "sleep apnoea" OR "sleep-disordered breathing" OR "sleep hypopnea" OR uvulopalatopharyngoplast* OR TI UPPP OR TI UP3 OR "upper airway stimulatation")

- Filters/limits: English
- Date searched: November 6, 2023
- # of records identified: 193

**TOTAL # of records identified through database searching:** 1,200 (including 541 duplicates)

**# of additional records identified through other sources:** 0

**# of records after duplicates removed:** 659
